# Tumor-derived exosomes induce CD8^+^ T cell suppressors

**DOI:** 10.1186/s40425-017-0269-7

**Published:** 2017-08-15

**Authors:** Brian T. Maybruck, Lukas W. Pfannenstiel, Marcela Diaz-Montero, Brian R. Gastman

**Affiliations:** 1 Department of Immunology, Lerner Research Institute, Cleveland, USA; 2 Institutes of Head and Neck, Dermatology and Plastic Surgery, Cleveland, USA; 3 Taussig Cancer Center, Cleveland Clinic, 9500 Euclid Ave/NE60, NE6-303, Cleveland, OH 44195 USA

**Keywords:** Exosomes, Suppressor, CD8 T cells, Galectin-1, RNA

## Abstract

**Background:**

The suppressive nature of immune cells in the tumor microenvironment plays a major role in regulating anti-tumor immune responses. Our previous work demonstrated that a soluble factor from tumor cells is able to induce a suppressor phenotype (SP) in human CD8^+^ T cells typified by loss of CD27/CD28 expression and acquisition of a potent suppressor function. The present study hypothesized that the soluble mechanism that is inducing the SP in CD8^+^ T cells are tumor-derived exosomes (TDEs).

**Methods:**

Membrane vesicles and TDEs from multiple head and neck cancer cell line’s conditioned growth media were isolated by ultracentrifugation and precipitation, respectively. Human purified CD3^+^CD8^+^ T cells were assessed for their induction of the T cell SP by flow cytometry identifying loss of CD27/CD28 expression and in vitro suppression assays. Furthermore, the T cell SP was characterized for the attenuation of IFN-γ production. To delineate exosomal proteins contributing to T cell SP, mass spectrometry was used to identify unique proteins that were present in TDEs. CRISPR/Cas9 knockout constructs were used to examine the role of one of these proteins, galectin-1. To assess the role of exosomal RNA, RNA purified from TDEs was nucleofected into CD8^+^ T cells followed by suppression analysis.

**Results:**

Using fractionated conditioned growth media, factors >200 kDa induced CD8^+^ T cell SP, which was determined to be an exosome by mass spectrometry analysis. Multiple head and neck cancer-derived cell lines were found to secrete T cell SP-inducing exosomes. Mass spectrometry analysis revealed that an immunoregulatory protein, galectin-1 (Gal-1), was expressed in those exosomes, but not in TDEs unable to induce T cell SP. Galectin-1 knockout cells were found to be less able to induce T cell SP. Furthermore, RNA purified from the T cell SP-inducing exosomes were found to partially induce the SP when transfected into normal CD8^+^ T cells.

**Conclusions:**

For the first-time, TDEs have been identified to induce a SP in CD8^+^ T cells and their mode of action may be synergistic effects from exosomal proteins and RNA. One protein in particular, galectin-1, appears to play a significant role in inducing T cell SP. Therefore, tumor-derived immunosuppressive exosomes are a potential therapeutic target to prevent T cell dysfunction and enhance anti-tumor immune responses.

## Background

Recent clinical successes of immunotherapies designed to boost anti-tumor immune responses in cancer patients has demonstrated the feasibility of using the immune system as a potent therapy. Indeed, the recruitment of tumor-specific cytotoxic CD8^+^ T cells to the tumor microenvironment is now understood to be generally associated with favorable clinical outcomes, even in patients not receiving immunotherapy [1, 2]. However, disease progression often occurs despite the presence of significant numbers of infiltrating immune cells, suggesting that immune recognition by itself is not sufficient for a productive clinical response. Recent studies have demonstrated that a large fraction of tumor-resident CD8^+^ T cells have phenotypic changes associated with exhaustion and dysfunction typified by the loss of expression of the immune co-receptors CD27 and CD28, as well as the expression of immune checkpoint inhibitor proteins such as programed death receptor 1 (PD-1), T cell immunoglobulin and mucin protein 3 (Tim-3), among many others [3–5]. T cells possessing these phenotypic changes are associated with tumor progression and poor clinical prognosis [6–8]. The mechanism by which the cytotoxicity of tumor-resident CD8^+^ T cells becomes blunted in the tumor microenvironment is incompletely understood, though it has been associated with the presence of significant populations of immune regulators including CD4^+^ regulatory T cells (Treg), and suppressive myeloid cells such as tumor-associated macrophages and myeloid-derived suppressor cells (MDSC) [9, 10].

Recent studies have also highlighted the secretion of tumor-derived exosomes (TDEs) as important contributors to the immunosuppressive tumor microenvironment [11]. Exosomes are cell-secreted vesicles containing proteins and nucleic acids from the parent cell, and are generally identified based upon their protein content (e.g., HSP70, CD63, β-Actin) and size (typically 50 to 100 nm in diameter); [12, 13]. Exosomes can transfer their contents (e.g., proteins and RNA) from one cell to another via fusion with the plasma membrane, and therefore are important regulators of the tumor microenvironment. Interestingly, exosomes released from tumor cells were demonstrated to carry factors that regulate immune responses, however little is known about the role of TDEs on the induction and activity of CD8^+^ T suppressor cells [14, 15].

We and others have previously reported that tumors can convert CD8^+^ T cells from cytotoxic effectors to inhibitors of anti-tumor immunity [16–18]. Specifically, we showed that tumor cell lines derived from various tissues directly induce phenotypic and functional changes associated with T cell dysfunction characterized by the loss of CD27 and CD28 expression and telomere shortening [16]. Furthermore, this process did not require direct contact with cancer cells and did not result from cell proliferation. We also demonstrated that treatment of T cells with cytoprotective cytokines such as IL-7 could resist the induction of suppression [16, 17].

In this report we show that CD8^+^ T cells isolated from the tumors of human head and neck cancer patients display the same dysfunction-associated phenotypic changes we previously found in tumor/T cell in vitro co-culture assays, especially a potent suppressive ability. Furthermore, we found that head and neck cancer cell line-secreted exosomes also induce a novel suppressive phenotype in CD8^+^ T cells. Upon demonstrating uptake of TDEs by T cells, we further show that these dysfunction-causing exosomes are secreted by a number of different head and neck cancer cell lines, and contain unique proteins, including galectin-1(Gal-1), which is known to play a role in immune regulation. We lastly demonstrate that RNA purified from exosomes is able to induce the suppressor phenotype when transfected into normal donor CD8^+^ T cells indicating that there is more than one factor responsible for the dysfunctional processes induced by TDEs. In summary, the release of TDEs contributes to the immunosuppressive nature of tumors by altering the function of CD8^+^ T cells resident in the tumor microenvironment, and implies that preventing or neutralizing TDEs may be a strategy to increase the efficacy of anti-tumor immune responses.

## Methods

### Tumor cell lines and T cells

Human colon carcinoma cell line Caco-2 and squamous cell carcinoma (SCC) of the head and neck cancer cell lines: Tu167, SCC0209, HN60 were cultured in complete DMEM (2 mM L-glutamine, 1% sodium pyruvate, 1% nonessential amino acids, 1% penicillin-streptomycin, and 10% membrane vesicle-free fetal bovine serum). The SCC0209 and HN60 cell lines were produced in-house from SCC tumor tissue obtained through and IRB-approved protocol. Briefly, tissue was dissociated by mincing into small fragments, which were cultured as described until cell monolayers were observed. Fibroblast outgrowth was removed by differential trypsinization. Tumor cells were passaged 6-10 times before use. Membrane vesicles from FBS were removed by overnight ultracentrifugation (100,000 × g) at 4 °C. Unless otherwise noted, CD3^+^ and CD3^+^CD8^+^ T cells were purified from PBMCs from normal volunteers by negative selection using the Pan T Cell Isolation and the CD8^+^ T Cell Isolation kits (Miltenyi Biotech), respectively. To ensure the purity of T cell populations (>95% purity) they were characterized by flow cytometry. Tumor-infiltrating lymphocytes were isolated after tumor digestion with Collagenase Type IV (750 units/ml final concentration; Gibco), Hyaluronidase (500 units/ml final concentration; Sigma-Aldrich), and DNAse I (2 mMU/ml) followed by Ficoll-Paque separation. CD8^+^ T cells were isolated from tumor cell suspensions using the Pan T Cell Isolation followed by CD8^+^ T cell positive selection beads or the CD8^+^ T Cell Isolation kits (Miltenyi Biotech).

### Isolation of tumor-derived membrane vesicles and exosomes

Tumor cell lines were grown to 70% confluence and conditioned growth media (CGM) was collected. CGM was centrifuged for 15 min at 3000 × *g* to remove cell debris. Membrane vesicles were isolated by overnight ultracentrifugation of the CGM at 100,000 × *g* at 4 °C. Next day, the supernatant was aspirated and the remaining pellet contained the membrane vesicle portion of the CGM. Exosomes were isolated from cell debris-free CGM using ExoQuick Exosome Precipitation Solution (System Biosciences) based on manufacturer’s instructions. Briefly, ExoQuick solution was added at a 1:5 dilution into CGM, inverted 10 times, and stored at 4 °C overnight. The following day exosomes were pelleted by centrifugation at 1500 × *g* for 30 min. Exosomes were then resuspended in 300 μl of sterile 1xPBS and measured for their protein concentration by BCA Protein Assay (Pierce).

### Identification of tumor-derived exosomal proteins

Based on protein concentration, an 8 μg aliquot from each exosome sample was subjected to overnight precipitation with acetone. The proteins were then reconstituted in 50 μl of 6 M urea, 100 mM tris digestion buffer. The protein concentration was reduced with DTT, alkylated with iodoaetamide, and digested overnight with trypsin. The LC-MS system was a Finnigan LTQ-Obitrap Elite hybrid mass spectrometer system. The HPLC column was a Dionex 15 cm × 75 μm id Acclaim Pepmap C18, 2 μm, 100 Å reversed- phase capillary chromatography column. Five μL volumes of the extract were injected and the peptides eluted from the column by an acetonitrile/0.1% formic acid gradient at a flow rate of 0.25 μL/min were introduced into the source of the mass spectrometer on-line. The microelectrospray ion source is operated at 2.5 kV. The digest was analyzed using the data dependent multitask capability of the instrument acquiring full scan mass spectra to determine peptide molecular weights and product ion spectra to determine amino acid sequence in successive instrument scans. The data were analyzed by using all CID spectra collected in the experiment to search the human, mouse, and bovine reference databases with the search programs Mascot and Sequest. The resulting search files were then uploaded into the program Scaffold for spectral count analysis.

### T cell suppression assays

Isolated T cells were cultured in complete RPMI with 30 μg/ml of purified exosomes for 6 h at 37 °C with 5% CO_2_. Controls included unfractionated tumor cell line CGM, non-exosome membrane vesicle CGM, and membrane vesicle-free complete RPMI. After incubation, cells were cultured for 7 days, harvested and then analyzed by flow cytometry for CD27/CD28 loss or used in suppression assays. For suppression assays T cells exposed to TDEs were co-cultured with un-manipulated (responders) isolated T cells from the same donor in culture plates coated with anti-CD3 (10 μg/ml) and soluble anti-CD28 (5 μg/ml) antibodies for 72 h. Wells were then either examined for Ki67 expression by flow cytometry or pulsed for the last 24 h with either 1 μCi (0.037 MBq)/well [^3^H]-thymidine or bromodeoxyuridine (BrdU). Thymidine incorporation was detected as described before. BrdU incorporation was detected by an indirect ELISA according to instructions from the manufacturer (EMD Millipore).

### Flow cytometry analysis of T cell surface molecules, IFN-γ, and Ki67

To determine T cell surface markers for suppressor phenotypes, T cells were labeled in flow cytometry buffer (FCB; 1× PBS buffer containing 2 mm EDTA and 0.5% BSA) with fluorochrome-conjugated anti-human CD3, CD4, CD8, CD27, and/or CD28 (Biolegend and BD Biosciences) for 30 min on ice in the dark. These cells were then fixed in 1% paraformaldehyde (PFA) overnight at 4 °C, washed and resuspended in FCB for flow cytometry analysis of these surface markers. To determine IFN-γ and Ki67 intracellular expression, T cells were first labeled with the surface fluorchrome-conjugated antibodies, CD3, CD4, and CD8 (BD Biosciences), and then permeabilized based upon Cytofix/Cytoperm (BD Biosciences) manufacturer instructions and then labeled with fluorochrome-conjugated anti-human IFN-γ and Ki67 (BD Biosciences). To assess surface and intracellular staining of antibodies samples were processed on a BD LSRFortessa or BD LSRFortessa X-20 (BD Biosciences) and analyzed with BD FACSDiva and FloJo software (FlowJo, LLC). Ten thousand to thirty thousand cell events were recorded, and dead cells and debris were excluded based upon lower forward scatter (FSC) and side scatter (SSC) signals (i.e., smaller size and granularity) followed by gating off of CD3^+^ cells. Identification of positive cell populations was based upon using FMO controls.

### Galectin-1 knockout and western blotting

Galectin-1 was knocked-out in Tu167 cells by co-transfection with Gal-1 specific CRISPR/Cas9 KO and HDR constructs obtained from Santa Cruz (sc-400,079 and sc-400,079-HDR) using Lipofectamine 2000 (Life Technologies) according to a previously reported protocol [19]. Cells were subjected to puromycin selection and single-cell dilution to generate clonal colonies, which were then screened for the absence of Galectin-1 expression by Western blot. Western blot analyses were performed as described previously [20]. Briefly, cell lines were seeded into 162 cm^2^ culture flasks (Corning). After reaching 70% confluence, cells were washed with 1× phosphate-buffered saline (PBS) and medium was replaced with Opti-MEM (Life Technologies). CGM was collected 48 h after media exchange and the exosome-containing fraction was isolated by ultracentrifugation as previously described. Total protein was isolated from the resulting pellets and from whole-cell cultures by extraction in radioimmunoprecipitation assay (RIPA) buffer containing protease inhibitors (Sigma). Proteins were separated by SDS-PAGE and transferred to a polyvinylidene fluoride membranes (Bio-Rad Laboratories). Primary antibodies used for blotting were anti-Galectin 1 (clone C-8, Santa Cruz Biotechnology), anti-HSP70 (clone W27, Biolegend), and Lamin A (clone H-102, Santa Cruz). Mouse anti-β-actin (clone C4, Santa Cruz) was used as a loading control. Horseradish peroxidase (HRP)-linked secondary antibodies were purchased from Cell Signaling Technology, and blots were developed using enhanced chemiluminescence (ECL) reagents from ThermoFisher or Advansta (Menlo Park, CA).

### Uptake of TDEs by CD8^+^ T cells and RNA nucleofection

Uptake of TDEs by CD8^+^ T cells was determined by staining TDEs with PKH67, a membrane labeling stain (Sigma-Aldrich) following manufacturer’s instructions. Stained TDEs were co-incubated with 1 × 10^6^ CD8^+^ T cells for 30 min and 2 h and analyzed by flow cytometry. For nucleofection experiments, RNA was isolated from purified exosomes by using Tri Reagent LS (Sigma-Aldrich) according to manufacturer’s instructions. Exosomal RNA was then introduced into freshly purified T cells by electroporation using a Nucleofector™ device following standard instructions (Amaxa). Nucleofected T cells were used in suppression assays as described above.

### Statistics

Means of all groups were compared for statistical differences by Student’s *t* test or a One-Way Analysis of Variance (ANOVA). A Bonferroni t test was used, following the ANOVA, to understand the statistical difference between two groups, when more than two groups were compared. Data was presented as means ± SD. Significance levels were set to *p* < 0.05.

## Results

### Tumor infiltrating CD8^+^ T cells from HNSCC patients show loss of CD27/CD28 and suppressive function

**Fig. 1:**
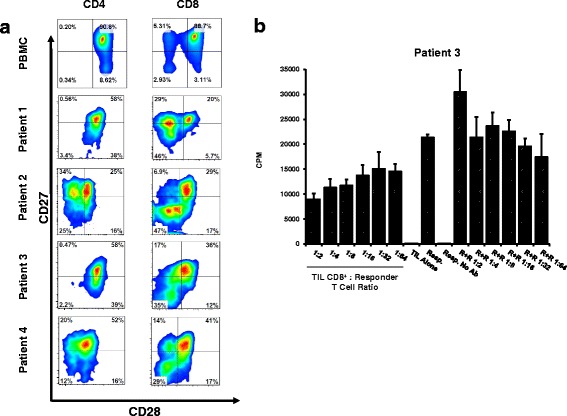
Loss of CD27/CD28 expression is present in tumor infiltrating CD8^+^ T cells from HNSCC patients and associates with suppressive function. **a**. Tumor tissue from four HNSCC patients was dissociated with collagenase/hyaluronidase before staining for FACS analysis. Plots show CD27 and CD28 expression on CD4^**+**^ (*left column*) and CD8^**+**^ (*right column*) T cells from each patient after gating on CD3^+^ cells. An age-matched donor PBMC stain is included for comparison. **b.** Tumor-infiltrating CD8^+^ T cells isolated from dissociated tumor tissue from Patient 3 were co-cultured with T cells isolated from peripheral blood (responders) from the same patient and proliferation in response to anti-CD3/CD28 stimulation was determined by Thymidine incorporation. R + R indicates wells where normal responder cells were used as negative control (non-suppressive) cells incubated with responders in similar ratios as the sorted TIL to verify decreased proliferation is due to suppression. Results are presented as average CPM ± SDEV at the indicated TIL:Responder ratio

Because loss of expression of CD27 and CD28 by T cells has been associated with suppressive function [16–18], we isolated CD3^+^ T cells from HNSCC tumors and determined their expression of CD27 and CD28. When compared to CD4^+^ T cells we found a significant fraction of CD8^+^ T cells lacking expression of both CD27 and CD28 in all 4 patients studied (Fig. 1a). In order to see whether CD8^+^ T cells, which lacked CD27/CD28 expression were also suppressive, we purified these cells from one patient via electronic sorting and used them in an *ex vivo* suppression assays using autologous peripheral blood T cells purified from the same patient as responder cells. We found that these tumor-infiltrating CD8^+^ T cells were able to suppress the proliferation of responder T cells after anti-CD3/CD28 stimulation (Fig. 1b). Note that despite the number of autologous responder T cells increasing, the suppression was still important from the CD8^+^ TIL between a ratio of 1:2 to 1:64. These results suggest that tumor infiltrating CD8^+^ T cells can contribute to the progression of tumors by directly inhibiting the normal function of neighboring T cells in the tumor microenvironment.

### Exposure to tumor derived factors >200 kDa induces a CD27^−^/CD28^−^ suppressive phenotype in CD3^**+**^ T cells

**Fig. 2:**
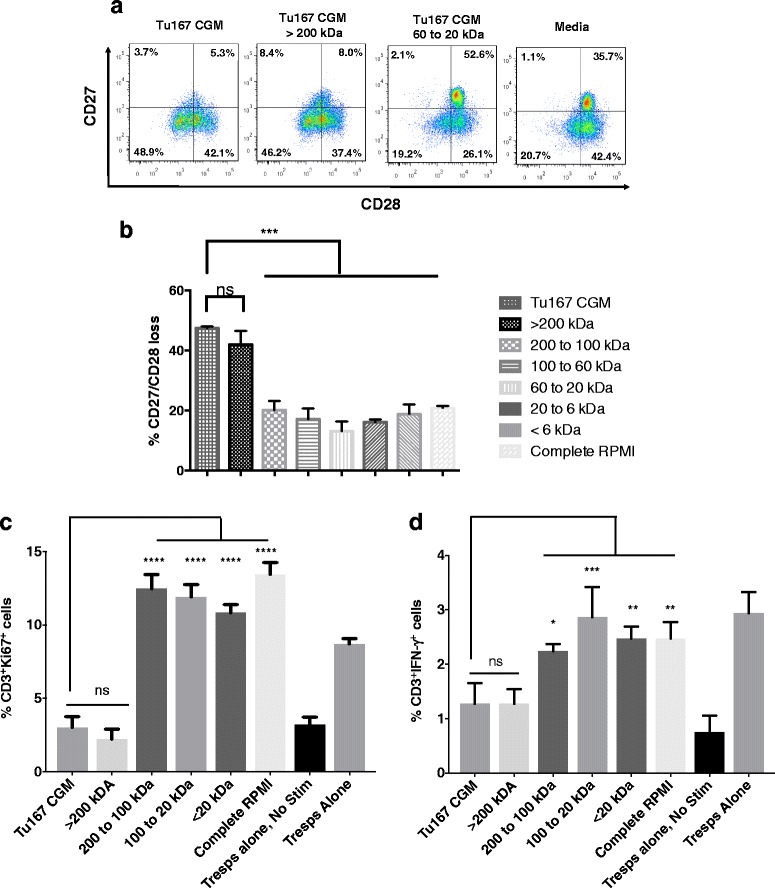
Serial fractionation of Tu167 conditioned growth medium (CGM) identifies that the >200 kDa fraction induces CD3^+^ T cells to have a suppressor phenotype: CD27/CD28 loss, suppression of responder T cell proliferation, and their reduction in the antitumor cytokine IFN-γ. After 70% confluence of the head and neck carcinoma tumor cell line Tu167, its CGM was spun down for 15 min at 3000 *x g* to remove cell debris. **a**. Tu167 CGM was first added to the ultrafiltration centrifuge tube that had the largest MWCO filter. Flow-through permeate was collected and added to the next smallest MWCO filter. This process was repeated until after the smallest MWCO filter. Retentate from each filter and permeate from smallest MWCO filter were then incubated with freshly purified T cells for 6 h. T cells were then washed and cultured for 7 days before staining for CD27/CD28 loss by flow cytometry. Dot plots are gated on CD3^+^. **b.** Bar graphs are the combination of five independent experiments and error bars represent ± SDEV. **c** to **d**. T cells were incubated under the above conditions and then mixed at a 1:2 ratio with autologous responder T cells for 3 days under anti-CD3/CD28 stimulation. Cells were then permeabilized and labeled with BV785, PE-Cy7, APC-R700-conjugated antibodies towards CD3, Ki67, and IFN-γ, respectively. Bar graphs represent the percent CD3^+^Ki67^+^ (**c**), and CD3^+^ IFN-γ^+^ (**F**) cells. Data represents the mean +/− SD of 3. To compare means, a one-way analysis of variance was used with a Bonferroni multiple comparison test. *p < 0.05, **p < 0.01, ***p < 0.001, and ****p < 0.0001 for the indicated groups vs. unfractionated conditioned media

We have previously reported that tumor cell-mediated induction of a dysfunctional CD27^*−*^/CD28^*−*^ suppressive phenotype in CD8^**+**^ T cells (CD8^**+**^ Ts) was not dependent on cell contact, and could occur when tumor and T cells were separated by 0.4 μm transwell inserts. These observations imply that the dysfunction-inducing factor was smaller than 0.4 μm [16, 17]. To further characterize this factor, we collected the CGM from cultures of Tu167 cells, a head and neck carcinoma tumor cell line used in our previous studies, and fractionated its contents using ultrafiltration centrifuge tubes that had progressively smaller molecular weight cut off sizes. The retentate from each tube was co-incubated with freshly purified normal donor CD3^+^ T cells for 6 h, followed by washing and culture for 7 days before assessing CD27 and CD28 loss. The fraction containing components larger than 200 kDa was able to induce significant CD27/CD28 loss, at a level comparable to that of unfractionated Tu167 CGM, while fractions containing smaller factors were unable to induce CD27/CD28 loss at all, and were comparable to media alone (Fig. 2a and b).

This suppressive phenotype caused by factors from CGM that were >200 kDa were further assessed in a suppression assay. CD3^+^ T cells where cultured under the previous indicated conditions and then examined for their ability to suppress autologous responder T cells by measuring the expression of the proliferation-specific nuclear protein, Ki67. As shown, unfractionated Tu167 CGM or the fraction of CGM greater than >200 kDa showed the ability to suppress the proliferation of responder T cells (Fig. 2c). Furthermore, when measuring the percentage of T cells producing IFN-γ, a cytokine known for its antitumor abilities, the Tu167 CGM fraction >200 kDa contributed to a significant decrease in the ability of responder T cells to produce IFN-γ (Fig. 2d) [21, 22]. Together, these data indicated that the dysfunction-inducing factor was >200 kDa.

### TDEs are important for the induction of CD3^**+**^ Ts cells

**Fig. 3:**
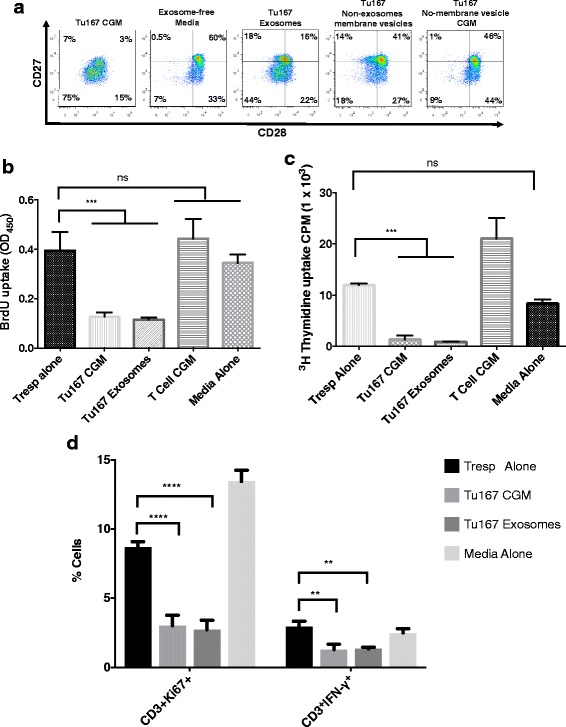
Tu167 tumor-derived exosomes are responsible for the induction of suppressor CD3^+^ T cells. Conditioned media from Tu167 cells had dead cells and debris removed by pelleting during centrifugation and aspirated supernatant was then incubated overnight at 4 °C with ExoQuick-TC (System Biosciences) to purify exsosomes. This produced an exosome fraction (pellet) and a non-exosome membrane vesicle fraction (supernatant). To get Tu167 non-membrane vesicle CGM fraction, Tu167 CGM was ultracentrifuged at 100,000 × g for 16 h at 4 °C. This produced an insoluble pellet (i.e., membrane vesicles) and soluble fraction (Tu167 No-membrane vesicle CGM fraction). After 6 h co-incubation with fractions, freshly purified CD8^+^ T cells were washed and grown in complete RPMI for 7 days. On day 7, a portion of CD8^+^ T cells were removed and prepared for flow cytometry. All dot plots are gated off of CD3^+^ lymphocytes. **a.** CD28 vs CD27 phenotype distribution for CD3^+^ T cells incubated under the indicated conditions. On day 6, a portion of CD8^+^ T cells were removed and used in in vitro suppression assays in anti-CD3- coated plates at a 2:1 responder: suppressor ratio. BrdU incorporation ELISA (**b**) and ^**3**^H-thymidine (**c**) suppression assay was done for 72 h with BrdU or ^**3**^H-thymidine being added 22 h before the end of the experiment. T cell CGM was collected from T cells purified from PBMCs and cultured in cRPMI for 3 days under anti-CD3/CD28 conditions. **d.** A suppression assay was also examined using KI67 and the antitumor cytokine IFN-γ at the above 2:1 responder: suppressor ratio with treatment of CD3^+^ T cells with exosomes. Data represents the mean +/− SD of 3. To compare means, a one-way analysis of variance was used with a Bonferroni multiple comparison test. *p < 0.05, **p < 0.01, ***p < 0.001, and ****p < 0.0001 for the indicated groups vs. responder T cells (Tresps) alone

We next sought to further characterize this >200 kDa fraction to identify whether the factor was a large protein, complex of proteins, or a membrane-bound vesicle. To separate possible vesicles from soluble proteins, CGM fractions were subjected to ultracentrifugation to pellet large membrane structures but not soluble proteins. The cleared supernatants were then analyzed for their ability to induce CD27/CD28 loss. Figure 3a demonstrates that removal of membrane vesicles abrogated the induction of CD27/CD28 loss. There is a variety of membrane vesicle related structures that tumors secrete. Given the growing literature on TDE’s we first assessed whether the secreted factor inducing CD27/CD28 loss was caused by exosomes. Using a widely-cited exosome-specific isolation kit, we isolated exosomes from Tu167-conditioned media. Figure 3a shows a large frequency of CD27/CD28 loss on normal CD3^**+**^ T cells after exposure to either Tu167 CGM or kit-isolated exosomes, but not non-exosome membrane vesicles. Loss of CD27/CD28 expression in both Tu167 exosome and Tu167 CGM-exposed T cells was accompanied by measuring the suppressive activity at a similar level as evidenced by using multiple suppression assay evaluation methods. We noted a decrease in BrdU (Fig. 3b **)** and thymidine incorporation (Fig. 3c **)**, and a decrease in Ki67 and IFN-γ expression (Fig. 3d) by normal responder T cells that had been incubated with T cells exposed to Tu167 TDEs and CGM. These findings indicate that TDEs can induce a regulatory phenotype in T cells, which are characterized by the loss of CD27/CD28 expression, the ability to suppress T cell responses to CD3/CD28 stimuli, and attenuate antitumor IFN-γ cytokine production from responder T cells.

### Induction of CD8^+^ Ts by TDEs can be found among multiple head and neck cancer cell lines

**Fig. 4:**
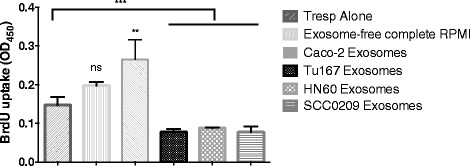
Tumor-derived exosomes from multiple cell lines are responsible for the induction of suppressor CD8^+^ T cells. CD8^+^ T cells were purified from human buffy coat and then incubated for 6 h with either TDEs from Tu167, head and neck cancer (HN60), squamous cell carcinoma (SCC0209) cells lines, or membrane-vesicle free complete RPMI (negative control). After incubation cells were washed and grown in exosome-free complete RPMI. On day 6, a portion of CD8^+^ T cells were removed and used for the above suppression assay at a 2:1, Responder T cells: CD8^+^ T cells. A BrdU incorporation ELISA suppression assay was done for 72 h with BrdU being added 22 h before the end of the experiment. Data represents the mean +/− SD of 3. To compare means, a one-way analysis of variance was used with a Bonferroni multiple comparison test. **p < 0.01, and ***p < 0.001 for the indicated groups vs. responder T cells (Tresps) alone

We then wanted to determine if exosome-mediated induction of CD3^+^ Ts could be delineated down to CD8^+^ Ts cells and was a phenomenon exclusively caused by Tu167 cells or whether it extended to other cell lines. To this end, exosomes were purified from the CGM of two other low-passage head and neck squamous cell carcinoma cell lines (developed from patients from the senior author) HN60 and SCC0209 in a manner similar to previous studies. Figure 4 shows that CD8^+^ T cells exposed to exosomes from HN60 or SCC0209 cells are able to suppress proliferation of normal T cells after CD3/CD28 stimulation at a similar level induced by exosomes isolated from Tu167 CGM. We further found that exosomes derived from Caco-2 cells (a human colorectal adenocarcinoma) were unable to induce both loss of CD27/28 (data not shown) and acquisition of suppressive function in normal T cells, indicating that not every cell line is capable of inducing this type of T cell dysfunction.

### The in vitro suppressive capability of TDE can induced CD8^+^ Ts cell formation at low concentrations and are regulatory beyond a Tresp: CD8^+^ Ts cell ratio of 512:1

**Fig. 5:**
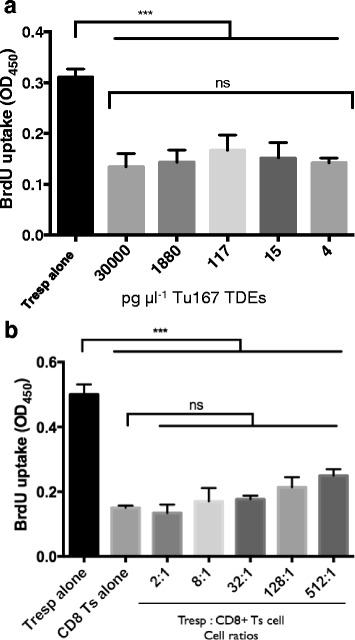
CD8^+^ T cells demonstrate the suppression of proliferation of responder T cells (Tresp) after incubating them with 4 pg μl ^−1^ TDEs and responder T cells: CD8+ T cells down to 512:1. **a**. CD8^+^ T cells were purified from human buffy coat and then incubated for 6 h with serially diluted concentration of 30,000 pg μl^−1^ of TDEs from Tu167. TDE concentrations were determined from BCA protein assay. After the CGM from the tumor cell lines had dead cells and debris removed, it was incubated overnight at 4 °C with ExoQuick-TC (System Biosciences) to purify exosomes. This produced an exosome fraction (pellet) and a non-exosome membrane vesicle fraction (supernatant). After incubation cells were washed and grown in complete RPMI for 7 days. On day 6, a portion of CD8^+^ T cells were removed and used for the above suppression assay at a 2:1, Responder T cells: CD8+ T cells. A BrdU incorporation ELISA suppression assay was done for 72 h with BrdU being added 22 h before the end of the experiment. **b**. The above was repeated with Tu167 TDEs but at 30 ng μl^−1^. On day 6, a portion of CD8+ T cells were removed and used for the suppression assay at ratios between 2:1 to 512:1, Responder T cells: CD8+ T cells. All data represents the mean +/− SD of 3. To compare means, a one-way analysis of variance was used with a Bonferroni multiple comparison test. ***p < 0.001 for the indicated groups vs. responder T cells (Tresps) alone

We have found that 30 μg ml^−1^ of TDE is an effective concentration for the induction of CD3^+^ Ts cells. To identify the role of TDEs as being physiologically relevant we examined the sensitivity of CD8^+^ T cells to the regulatory-inducing effects of TDEs. We discovered that regardless of the greater than 7000 fold difference between the highest and lowest TDE concentration used to induce the formation of CD8^+^ Ts cells, all responder T cells had their proliferation equally suppressed (Fig. 5a). The concentrations of TDEs used to induce CD8^+^ Ts cell formation equated to a range of 4 fg cell^−1^ to 30 ng cell^−1^. Overall, this implies that CD8^+^ T cells are sensitive to relatively low concentrations of TDEs within the range that we tested.

To identify the proliferative and suppressive capability of CD8^+^ Ts cells that were induced by TDEs, CD8^+^ Ts cells were cocultured at increasing ratios and stimulated with plate-bound anti-CD3 and soluble anti-CD28. CD8^+^ Ts cells demonstrated the characteristic in vitro hypoproliferation when cultured alone. When CD8^+^ Ts cells were cocultured with Tresps, regardless of the increasing cell ratios between the two cells, there was minimal change in T cell proliferation when compared to CD8^+^ Ts cells incubated alone. At the lowest (2:1) and highest (512:1) cell ratios (Tresps: CD8^+^ Ts cells) the suppressive capabilities of CD8^+^ Ts cells on the proliferation of Tresps was 73% and 50%, respectively (Fig. 5b).

### CD8^+^ T cells interact with TDEs

**Fig. 6:**
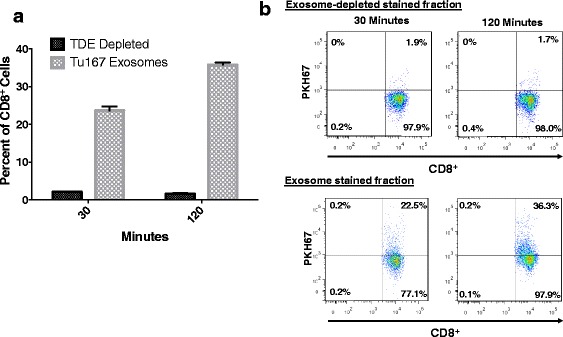
CD8^+^ T cells can uptake tumor-derived exosomes. 3 μg (1.5 μg/ml) of Tu167 TDEs were labeled with PKH67 dye. The flow-through produced during the concentration of the labeled TDEs, and the labeled TDEs were incubated with 1 × 10^6^ CD3^+^CD8^+^ T cells for 30 and 120 min. Unbound exosomes were removed from cells by multiple washes with flow cytometry buffer (1× PBS, 2 mM EDTA, and 0.5% BSA). **a**. *Dot plots* above were first gated off of CD3^+^ T cells and then gates were set to CD8^+^ T cells vs. PKH67 negative (T cells not incubated with labeled TDEs). **b**. Cells positive for TDE uptake are characterized as being double positive for CD8^+^ and PKH67. Data represents the mean ± SD of 3 replicates

To better characterize the interactions between TDEs and CD8^+^ T cells, exosomes derived from Tu167 cells were treated with the membrane labeling stain PKH67. Stained exosomes were co-incubated with purified normal human donor CD8^+^ T cells for 30 or 120 min. CD8^+^ T cells were then washed, and dye uptake was verified by flow cytometry. Figure 6a shows 22.5% and 35.6% of purified CD8^+^ T cells positive for PKH67 after 30 and 120 min of co-culture, respectively. To rule out non-specific uptake of PKH67, exosome-depleted stained fractions were included. Incorporation of PKH67 was significantly higher in CD8^+^ T cells incubated with labeled exosomes than in CD8^+^ T cells incubated with exosome-depleted stained fractions (Fig. 6b). These results reveal that CD8^+^ T cells are able to interact with exosomes secreted by tumor cells, and suggest that exosomes containing dysfunction-inducing factors are able to connect with T cells in the tumor microenvironment.

### Dysfunction-inducing TDE contain galectin-1 (Gal-1), a protein associated with immune modulation

To identify candidate proteins from the TDEs that may be important in the induction of CD8^+^ Ts cell formation, and to confirm that the isolated vesicle fraction indeed contains exosomes, purified TDEs from Tu167, HN60, Caco-2, and SCC0209 were precipitated and digested overnight with trypsin. The digests were then analyzed by capillary column LC-tandem MS and the CID spectra searched against a human reference sequence database. For a protein to be matched to the human database it had at least one unique peptide. Table 1 presents the spectral counts of a number of proteins that previous studies have demonstrated to be present in exosomes derived from a wide variety of cancers [12]. While not every protein was detected in the exosomes from the cell lines, each fraction contained at least two known exosome-associated proteins.

**Table 1:** Confirming isolation of TDEs

Protein	Accession no.	MW	Tu167 TDE Peptide #	HN60 TDE Peptide #	SCC0209 TDE Peptide #	Caco2 TDE Peptide #
Heat shock protein 70	194248072	70	12			
Glyceraldehyde 3-phoshpate dehydrogenase	7669492	36	15	2	6	6
Heat shock protein 90	153792590	98	7		5	8
Annexin A2	209862831	39	17		13	14
Moesin	4505257	68	5			
Pyruvate Kinase	33286418	58	26		14	11
Alix	22027538	96	24			
Elongation factor1 alpha	4503471	50	16		7	8
Cofilin 1	5031635	19	16	2	2	13
Peptidylproplyl isomerase A	10863927	18	12		2	9
Ezrin	21614499	69	12			9
Phospho-glycerate kinase 1	4505763	45	6			

Further analysis of results of the MS study resulted in 152, 60, 137, and 150 exosomal proteins being identified in TDEs from Tu167, HN60, SCC0209, and Caco-2 respectively. These lists were refined into one list of proteins that not only mutually occurred among the different TDEs, but also were greater than moderately expressed (Table 2). This produced a list of 15 proteins of which all but one protein could be considered to be involved in cell adhesion, migration, and structure: galectin-1 (Gal-1). This protein is well-documented for its immunomodulatory role in regulating immunogenic T cell effects.

**Table 2:** TDE proteins that are greater than moderately expressed and shared between different cells that cause the induction of suppressor T cells

Protein	Accession no.	MW	Tu167 TDE Spectral Counts	HN60 TDE Spectral Counts	SCC0209 TDE Spectral Counts	Caco2 TDE Spectral Counts
Collagen alpha-1(XII) chain long isoform precursor	93141047	333 kDa	10	13	223	5
EGF-containing fibulin-like extracellular matrix protein	86788015	55 kDa	20	11	49	0
Fibrillin-1	281485550	78 kDa	16	26	190	0
galectin-1	4504981	15 kDa	14	14	25	0
Latent-transforming growth factor beta-binding protein 2	4557733	195 kDa	32	17	58	0
Thrombospodin-2	40317628	130 kDa	125	11	82	0
Transforming growth factor-beta-induced protein ig-h3 precursor	4507467	75 kDa	83	67	169	6

**Fig. 7:**
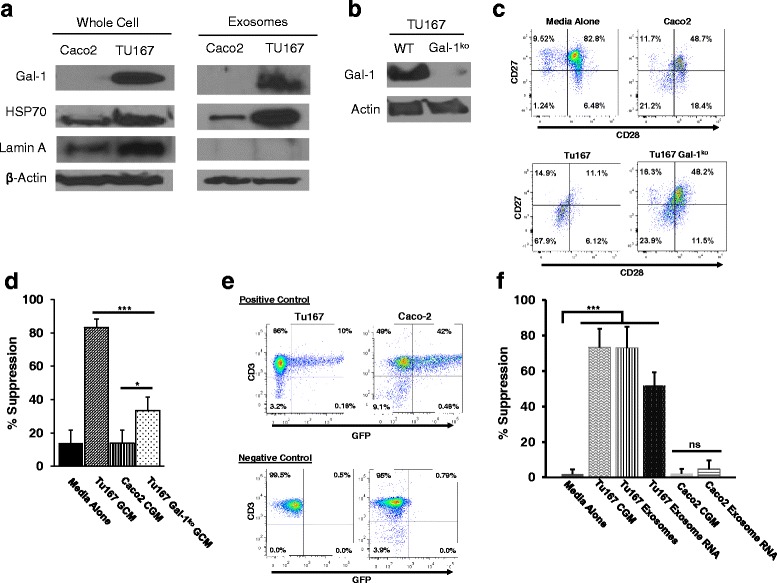
Exosomal galectin-1 and RNA factors contribute to the induction of CD8^+^ Ts cells. **a**. Conditioned media from Tu167 and Caco-2 cells was harvested after 48 h, centrifuged and filtered to remove cellular debris, and then ultracentrifuged for 18 h to pellet the exosome fraction. Protein was isolated by extraction in RIPA buffer and used in western blotting for the indicated proteins. For comparison, whole cell lysates were prepared from the same cell lines. **b.** Galectin-1 was then knocked-out in Tu167 cells by CRISPR/Cas9 targeting constructs, which was then verified by Western blot of whole-cell lysates. **c**. Conditioned growth media from Tu167 wildtype and Gal-1^ko^ cells, as well as Caco-2 control cells was then collected as previously described, and exosomes were enriched via ultracentrifugation and incubated with normal donor T cells for 6 h. Cells were then washed and cultured for an additional 6 days before assessing for CD27 and CD28 expression on CD8^+^ T cells by flow cytometry. Indicated plots are gated on live CD3^+^ CD8^+^ cells. **d**. Treated CD8^+^ T cells were then suppression assays with CFSE-labeled, normal donor T cells as responders similar to previous experiments. **e.** RNA was isolated from purified exosomes by Trizol extraction and introduced into freshly purified T cells by electroporation using a Nucleofector™ device following manufacturer’s instructions (Amaxa). Successful Nucleofection was verified by the addition of a GFP-expressing plasmid followed by analysis by flow cytometry. Nucleofected T cells were then incubated for 6 days and then used in an in vitro suppression assay as previously described. **f**. ^**3**^H-thymidine incorporation suppression assay was done for 72 h with ^**3**^H-thymidine being added 22 h before the end of the experiment. All data represents the mean +/− SD of 3. To compare means, a one-way analysis of variance was used with a Bonferroni multiple comparison test. *p < 0.05, ***p < 0.001 for the indicated groups vs. incubation of T cells in cRPMI followed by incubation with responder T cells at 1:2 ratio

In order to confirm that Gal-1 is expressed in exosomes produced by Tu167 cells (Ts inducing), but not in those derived from Caco-2 cells (not Ts inducing), we performed Western blot analysis on protein harvested from tumor cell-conditioned media following ultracentrifugation (Fig. 7a **)**. These blots confirm that Gal-1 protein is absent in fractions isolated from Caco-2 cells, but present in those derived from Tu167. Interestingly, similar blots of whole-cell extracts also found an absence of Gal-1 protein in Caco-2 cells, indicating that its absence is due to a lack of expression in this cell type. To confirm that the protein from conditioned media did not come from whole-cell lysates, we also blotted for proteins commonly known to be present in exosomes (HSP70 and β-actin) and one that should be absent in exosomes (Lamin A, a nuclear envelope protein). These blots demonstrated that HSP70 and β-actin were both present in whole cell and exosome extracts from TU167 and Caco-2 cells, while Lamin A was only detected in the whole-cell extracts from both cell lines. These data confirm that conditioned-media fractions did not contain protein from whole-cells.

In order to further examine the role of galectin-1 in the induction of Ts, we then used CRISPR/Cas9 targeting constructs to knock-out galectin-1 in Tu167 cells. After transfection and selection, a clone lacking galectin-1 expression was selected for further studies after verification of galectin-1 knockdown by Western blot (Fig. 7b). Conditioned growth media from these cells was then used to induce Ts in normal donor CD8^+^ T cells as compared to media conditioned by wildtype Tu167 and Caco-2 (a non-Ts inducing control) cells similar to preceding studies. We found that conditioned media (containing TDE) from the galectin-1 knockout cells were significantly less able to induce loss of CD27/CD28 expression, though not completely (Fig. 7c). We then used these cells in suppression assays with normal responder T cells from the same donor, and found that the knockout of galectin-1 expression also reduced the suppressive ability of CGM-treated CD8^+^ T cells (Fig. 7d). Together, these data suggest a role for galectin-1 in the induction of Ts-phenotype and functional changes by tumor-derived exosomes. However because the reversal was not complete, multiple factors may be present in TDE that optimally induce Ts phenotypic changes.

### RNA contained within TDEs contributes to the generation of CD8 Ts

Recent studies have demonstrated that tumor-derived exosomes also contain mRNAs in addition to proteins, and the presence of these RNAs can have an impact on the function of nearby cells. [12, 15] With this in mind, we next sought to determine whether RNAs contained within TDE were able to contribute to the induction T cell dysfunction. More than 2 μg of RNA was isolated from Tu167 and Caco-2 exosomes via TRIzol extraction, and transfected into normal T cells along with plasmid DNA encoding GFP in order to visualize the efficiency of transfection. Figure 7e shows that T cells transfected with the GFP construct are able to express the marker. These transfected T cells were then used in co-culture suppression assays with normal responder T cells from the same donor. We found that T cells transfected with RNA isolated from Tu167 exosomes induced T cells to become suppressive similar to both conditioned media and purified exosomes. Similar to previous studies, exosomal RNA isolated from Caco-2 cells was also unable to induce the acquisition of suppressive function (Fig. 7f). Together, these data suggest that the dysfunction-inducing factors contained within Tu167 exosomes may be due to RNAs as well as proteins, and that several mechanisms may cause dysfunctional changes in T cells present in the tumor microenvironment.

## Discussion

The recent clinical successes of immune-based cancer therapies highlight the utility of harnessing the cytotoxicity of CD8^**+**^ T cells to target tumors. While current immunotherapies are effective at supporting and enhancing the function of tumor-specific T cell responses, these cells remain subject to multiple tolerizing mechanisms in the tumor microenvironment which can ultimately limit their effectiveness. While the function of dedicated regulatory immune cell populations like MDSCs and Tregs are well-studied contributors to tumor tolerance, our studies suggest that additional tumor-mediated mechanisms exist.

Our previous studies indicated that tumor cell lines secreted a soluble factor that was capable of inducing phenotypic and functional changes in normal human T cells that closely resembled those associated with dysfunctional T cells present in the periphery and tumor microenvironment of cancer patients [16, 17]. Because these studies were based on in vitro co-culture experiments, we first sought to identify whether the same CD27^−^/CD28^−^ cells also observed in the tumor microenvironments of head and neck cancer patients were also suppressive. Indeed, these cells were able to suppress the proliferation of peripheral T cells isolated from the same patient. These data suggest that the soluble dysfunction-inducing factor observed in our previous studies could contribute to the immune-regulatory nature of the tumor microenvironment.

In order to characterize the mechanisms behind the induction of the regulatory phenotype in these CD8^**+**^ T cells, we explored the contents of media conditioned by multiple cancer cell lines. We show that TDEs are important in inducing CD8^**+**^ Ts cells. Membrane vesicles can include apoptotic bodies, microvesicles, and exosomes, with exosomes being the smallest at between 50 to 100 nm in diameter [23]. Previously, TDEs have been implicated in the induction of self-tolerance associated with CD8^**+**^ T cells, but their role in the induction of CD8 Ts has not been reported before. Exosomes from human prostate cancer, in a dose-dependent manner, via TDE expression of FasL, caused CD8^**+**^ T cells apoptosis [14]. In another example, multiple tumor cells lines demonstrated their selection towards removal of MHC-I MICA and MICB ligands by packaging them into exosomes to avoid their recognition by the NKG2D activating receptor found on patrolling cytotoxic CD8 T cells and natural killer cells [24]. In addition, TDEs containing TGF-β were shown to cause the down-regulation of the NKG2D receptor on CD8^**+**^ T cells, preventing their activation [11].

To test our hypothesis, that TDEs play a role in the induction CD8^**+**^ Ts cells, TDEs were purified from Tu167 CGM by using a widely-cited exosome precipitation solution [25–29]. Confirmation that this method was able to purify exosomes was based upon comparing the purified exosome’s protein profile to an exosomal protein database. From the proteomics analysis of Tu167 TDEs we are able to detect 10 commonly found proteins in exosomes. This included heat shock protein 70 and glyceraldehyde 3-phosphate dehydrogenase that are the first and third most common proteins found in exosomes, respectively [12]. To further confirm that we had purified exosomes, we looked for proteins that should not be present in purified exosome fraction based on their intracellular membrane-bound organelle locations. Proteins that have been used previously for this purpose are: GM130, calnexin, and cytochrome C [30]. According to our exosomal protein profile none of those proteins were identified, confirming the purity of our purified exosomes.

To explore the role of protein components of the TDEs on the induction of CD8^**+**^ Ts cells, we compared the protein content of exosomes that do and do not induce CD8^+^ T cell dysfunction. The similarity of proteins being identified by capillary column LC-tandem MS, and greater than moderately expressed as exosomal proteins among the tumor cell lines used (i.e., Tu167, HN60, and SCC0209), revealed 15 proteins. Of these, Gal-1 is associated with immunoregulatory functions. Interestingly, we found that Gal-1 was present in cell lines able to induce Ts T cells and absent in those that are not. Furthermore, when we knocked-out Gal-1 in Tu167 cells, a line previously able to induce Ts, we found that TDE from these cells were significantly less able to induce both loss of CD27/CD28 expression and suppressive function.

Galectins are from the family of lectins that are well-documented in being a part of regulating innate and adaptive immunity [31]. Specifically, Gal-1 was shown to be involved in tolerogenic effects. For example, the neutrophil and mast cell inflammatory response is impaired by Gal-1 by preventing their extravasation [32]. In the context of the adaptive immunity, Tregs were shown to be expanded and functionally suppressive when co-incubated with Gal-1 [33]. Furthermore, membrane-bound Gal-1 expressed by Tregs is a ligand for the GM1 receptor on activated CD4^**+**^ and CD8^**+**^ T cells, causing these effector T cells to become suppressed in their proliferation [34]. That study did not examine if CD8^**+**^ T cells that were suppressed by Gal-1 were also induced to become suppressive. Clearly, these findings as well as our novel identification of Gal-1 as a player in the induction of CD8^+^ Ts cells indicates that Gal-1 requires further characterization.

That the knock-out of galectin-1 by itself was unable to completely eliminate the ability of TDE to induce CD8^+^ Ts cells suggests that there may be multiple factors that contribute to the process. A further characterization of exosome contents revealed that in addition to protein, they can also contain RNAs. Since TDE embedded RNAs can have an impact on the function of nearby cells [12, 15], we explored the importance of TDE RNA as a mechanism causing the induction of Ts cells. Our data show that RNA isolated from Tu167 TDEs does induce the suppressor phenotype in CD8^**+**^ T cells. This implies that both proteins and RNAs derived from tumor cells may play a role in inducing dysfunction in nearby T cells. Although the literature does not describe TDEs RNA causing the induction of suppressive CD8^**+**^ T cells, TDE RNA has been observed to induce an important immunomodulatory role. MicroRNA from pancreatic cancer derived exosomes was shown to induce tolerance by preventing the expression of MHC II from dendritic cells [21]. Furthermore, effector T cells were found to be suppressed in their proliferation due to miRNA when co-incubated with Treg exosomes, but not when co-incubated with exosomes from dicer deficient Tregs [22]. Glibolastoma TDEs horizontally transferred mRNA into cells that induced glioma cells tumorigenesis [35]. Clearly, additional studies are required to identify specific mRNAs or miRNA species within dysfunction-inducing exosomes and characterize their effects on the process of CD8^+^ Ts cell induction.

Due to the potential presence of tumor-derived exosomes in the blood, and the unique biomarkers associated with them, TDEs provide the opportunity for not only real-time, non-invasive characterization for diagnosis and prognosis of specific cancer types, but also their use as a vaccine [36, 37]. Murine dendritic cell in vitro uptake of TDEs has been shown to induce a pro-inflammatory phenotype in DCs, and after their addition to myeloid leukemia (WEHI3B) bearing mice, hyper-proliferation of lymph node cells was noted along with greater survival time of mice [38]. Interestingly in this study, Caco2 TDEs induced a hyper-proliferative response in T cells suggesting that these TDEs, unlike others that help promote tumor growth (e.g., suppress immune response, induce angiogenesis; 12), might be a hindrance to Caco2 survival within a host. Previous work has suggested that TDEs might be useful as nanoscale particles inducing a protective immune response against cancer [39]. This could imply that Caco2 TDEs could be studied for their usefulness as a vaccine providing protection from colorectal carcinomas.

## Conclusions

Understanding the unique phenotype of TDEs and their role in promoting tumor progression will provide potential targets for therapeutic intervention. Our study illustrates that TDEs are a mechanism used by multiple tumor cell lines to induce the formation of CD8^**+**^ Ts, especially through the presence of galectin-1 protein. Due to access to head and neck cancer patient tissue and blood our focus was in this cancer type. More work into other cancer types will be essential to best understand the full implications of TDE induced CD8^+^ Ts cells, as well as identifying the role that proteins such as galectin-1 and the RNA content of TDE play in this process. Given that these CD8^**+**^ Ts cells are identified in the tumor microenvironment of actual patients at high levels, blocking TDEs should have a high potential in improving immune-based therapies. Thus, greater understanding of the role of TDE’s in tumor induced immune dysfunction should lead to the discovery of new strategies of efficacious cancer immunotherapy.

## Abbreviations

BrdU: Bromodeoxyuridine
Caco-2: Human colon carcinoma cell line
CGM: Conditioned growth media
ECL: Enhanced chemiluminescence
FBS: Fetal-bovine serum
Gal-1: Galectin-1
MDSC: Myeloid-derived suppressor cells
PBS: Phosphate-buffered saline
PD-1: Programed death receptor 1
RIPA: Radioimmunoprecipitation assay buffer
SP: Suppressor phenotype
TDEs: Tumor-derived exosomes
Tim-3: T cell immunoglobulin and mucin protein 3
Treg: CD4^+^ regulatory T cells
Ts: Suppressor T cells
Tu167, SCC0209, and HN60: Head and neck cancer cell lines
